# Passive Millimeter-Wave Imaging for Burns Diagnostics under Dressing Materials

**DOI:** 10.3390/s22072428

**Published:** 2022-03-22

**Authors:** Amani Yousef Owda

**Affiliations:** Department of Natural, Engineering and Technology Sciences, Arab American University, Ramallah P600, Palestine; amani.owda@aaup.edu

**Keywords:** burns, millimeter-wave, passive imaging, noncontact diagnostics, dressing materials, porcine skin

## Abstract

This paper presents a feasibility study of using a passive millimeter-wave imaging (PMMWI) system to assess burn wounds and the potential for monitoring the healing process under dressing materials, without their painful removal. Experimental images obtained from ex vivo porcine skin samples indicate that a ThruVision passive imager operating over the band 232–268 GHz can be used for diagnosing burns and for potentially monitoring the healing under dressing materials. Experimental images show that single and multiple burns are observed throughout dressing materials. As the interaction of millimeter-wave (MMW) radiation with the human body is almost exclusively with the skin, the major outcomes of the research are that PMMWI is capable of discriminating burn-damaged skin from unburned skin, and these measurements can be made through bandages without the imager making any physical contact with the skin or the bandage. This highlights the opportunity that the healing of burn wounds can be assessed and monitored without the removal of dressing materials. The key innovation in this work is in detecting single and multiple burns under dressing materials in noncontact with the skin and without exposing the skin to any type of manmade radiation (i.e., passive sensing technology). These images represent the first demonstration of burns wound under dressing materials using a passive sensing imager.

## 1. Introduction

**Figure 1:**
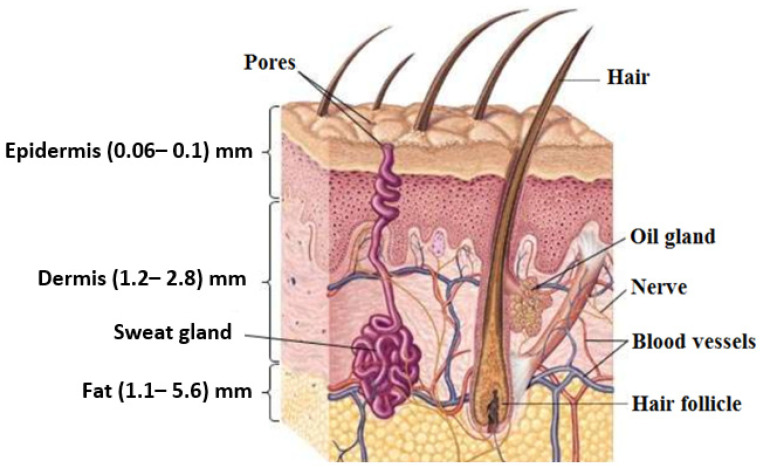
The human skin structure and its derivatives (sweat glands, oil glands, nails, hair, and hair follicles) [15].

Human skin has three main layers, namely, the epidermis (outer protective layer), the dermis (inner connective layer), and the hypodermis (subcutaneous fat layer), as illustrated in Figure 1. For adults, skin covers a total surface area of ~1.7 m^2^ and constitutes ~15% of the total body weight [1,2]. Human skin is different compared with other organs; it presents our interface to the external world, it is the first part of the human body that can be seen by others, and it provides an indication about the state of health of the human body through the appearance of the skin [3]. During the cycle of life, human skin is affected by many factors, such as age, the environment, the interaction with different types of radiation, genetic defects, and accidents. These factors might cause diseases, temporal skin conditions, and permanent disorders.

A burn is a thermal injury or tissue damage that results from thermal, chemical, and electrical traumas, and excessive sun exposure [4]. Based on the depth of invasion, burns are classified into partial thickness and full thickness [5]. In medical practice, the treatments of burn injury are based on the degree of the burn, the severity of the burn, and the medical circumstances of the patient. As an example of this, the first-degree burn can be treated using cold water, pain relief, creams, and bandages that are used to facilitate the wound healing process [6]. Alternatively, the third- and the fourth-degree burns require different treatments that might include surgery (surgical excision and grafting), rehabilitation, physical therapy, and lifelong assisted care [7].

Visual inspection is the current medical practice for assessing burn wounds [8,9]. This practice involves the removal of dressing materials that might be uncomfortable and painful to the patient [8]. As an alternative to the current medical practice, millimeter-wave imaging technology is suggested for use [10] due to (1) non-ionizing nature [11], (2) capability of providing highly localized measurements for the skin [12], (3) sensitivity to variations in water content in biological tissues caused by burns wound [10,13], and (4) capability of penetrating dressing materials and clothing [14].

In medical applications, images over the millimeter-wave (MMW) band can be implemented passively; where the natural thermal radiation emitted and reflected by the human body is used [16], or actively using radar; where the transmitter provides artificial MMW radiation to illuminate the human body, and the image is formed from the reflected radiation [10,17]. Passive millimeter-wave images are free from artefacts such as speckle and glint as the illuminating radiation from the human body and the environment is spatially incoherent [18,19]. Therefore, it is used in this paper to assess the feasibility of using PMMWI for detecting burns under dressing materials.

In general, the main elements of the PMMWI system are (1) high-speed scanner, (2) multiple receivers, (3) a high-gain antenna, (4) focusing elements such as mirrors and lenses, and (5) data processing software with a display [20]. The PMMWI consists of multiple independent channels of a single channel receiver. These channels gather the data to be combined into a single image [20,21].The minimum detectable radiation temperature variation $\Delta T_{min}$ for a radiometer (thermal sensitivity) is given by the radiometer equation, [22,23,24,25] namely,

(1) $$\Delta T_{min}=\frac{T_{A}+T_{R}}{\sqrt{B\;t}}$$

where *t* is the post-detection integration time, *T_R_* is the receiver noise temperature, *B* is the receiver bandwidth, and *T_A_* is the antenna radiation temperature, effectively, the radiation temperature of the source in front of the antenna [24].

### Related Work

The MMW band is the electromagnetic region between the microwave and terahertz, covering the frequency ranges 30–300 GHz [26,27,28]. Radiation in this band is known as a millimeter-wave since the wavelength of this radiation lies between 10 mm and 1.0 mm [28]. MMW radiations are capable of penetrating dressing materials and they are very sensitive to the variations in water content in biological tissues that might be due to the immediate response of burn injury [29]. Therefore, they have been suggested to be used for burns wound diagnostics.

For active sensing technology, an active millimeter-wave imaging scanner having a center frequency of 94 GHz [10] was used to scan images of the healing of the scars of the human hand under the plaster of Paris using an in-contact active scanner. Refs. [4,30] suggested synthetic aperture radar (SAR) for demonstrating features and burns of porcine skin samples under dressing materials. Ref. [31] used monostatic radar for measuring the optical path length of dressing materials over the frequency band 15–40 GHz. These studies [4,10,30,31] demonstrate the capability of active sensing technology to penetrate dressing materials and provide useful information about the skin and burns under dressing materials. However, this technology used artificial manmade radiation to form images. Although these radiations are non-ionizing and it is believed that they are not harmful [32,33], for medical applications purposes, it is recommended to use passive sensing technology as it does not expose the human body to any type of radiation.

For passive sensing technology, a simulation model in [12] was used to investigate the differences in the mean emissivity values between healthy skin and diseased skin over the MMW band 30–300 GHz. The model suggested that radiometry can be used as a noncontact sensor to distinguish between healthy skin and diseased skin. A single channel radiometer in [8] was used to measure the emissivity of a phantom chicken before and after the application of localized heat treatment. The main findings of this research [8] are that there are substantial differences in the mean emissivity values between unburned and burn damaged skin. MMW reflectometry in [30] suggested that an open-ended coaxial probe can be used as a noninvasive in-contact technique to distinguish between unburned and burned skin having different degrees of burn injuries. Radiometric measurements in [29] showed a clear signature for the burn-damaged skin and suggested that radiometry can be used as a noncontact sensor to determine the severity of the burns and the degree of the burns. Overall, these publications [8,12,29,30] focus on measuring the mean emissivity values (quantitative measure) of the skin and the signature of unburned and burn-damaged skin. However, this paper presents experimental images obtained from porcine skin samples before and after the application of localized heat treatment with and without the presence of dressing materials using PMMWI system. The key innovation in this work is in detecting single and multiple burns under dressing materials in noncontact with the skin and without exposing the skin to any type of manmade radiation (i.e., passive sensing technology). These images represent the first demonstration of burns wound under dressing materials using a passive sensing imager. The next paragraphs provide overviews of emerging technologies that are suggested to be used for assessing burn wounds, such as terahertz imaging, optical coherence tomography, and infrared imaging.

Terahertz frequency band (THz > 300 GHz) is another technology suggested to be used for the noninvasive diagnosis of burn wounds as it proves the potential for measuring burn depth, which is hard to monitor and assess clinically [34]. The terahertz pulse imaging in [35] showed that burn scars provide a well-defined contrast in reflectance compared with healthy tissue. Images from second- and third-degree burn wounds from rats in [36] indicated that THz time-domain spectroscopy had the capability to distinguish between partial-thickness and full-thickness burn wounds. The in vivo study in [37] showed the dynamic variations in skin reflectivity after the burning process; experimental measurements showed that burned skin had higher reflectivity as a result of increased water concentration due to the post-injury inflammatory response, whereas unburned skin showed a lower reflectivity. Although terahertz imaging seems to be a promising technology for burn wounds, the low penetration depth of THz radiation is the main limitation of this technology.

Optical coherence tomography (OCT) is suggested to be used for the noninvasive diagnosis of burn-damaged skin due to its capability of providing a high resolution of ~10 µm. The study in [38] suggested that polarization-sensitive optical coherence tomography (PS-OCT) is feasible to assess the burn wound depth in the first two days only as increasing the water content and exudates due to edema formation present the main limitation. Ref. [39] showed high-resolution cross-sectional images obtained from the high-speed fiber-based PS-OCT; the images showed that the detectable amount of collagen content in the burning surface is useful to assess the degree of the burn, as severe burns have higher collagen content compared with superficial burns. Ref. [40] indicated that the three-dimensional images obtained from PS-OCT can characterize the burn wound based on vasculature and birefringence. The study in [41] used spectroscopic optical coherence tomography (SOCT) and showed significant differences in the spectra associated with the depth of the burn. The conventional OCT has a spatial resolution of ~10 µm recorded at a penetration depth of 1.0 mm of the tissue [42]. According to Rayleigh [43,44], the main limitation of OCT is the scattering that is inversely proportional to the wavelength, and this makes the phase reconstruction of an image at depth through the overlying tissue difficult.

In the infrared frequency band, an infrared camera [45] was used to examine the healing progress of the burn wound by estimating the surface area of the burn based on temperature detection. The study in [46] presented thermographic images obtained from patients having burns injury. Experimental results in [46] indicated that deep burns expressed a significant variation in the temperature after the burn injury and these results are in good agreement with the results obtained from another study conducted on pigs [47]. The active dynamic infrared thermal imaging in [48] showed the ability of active dynamic infrared thermal imaging to assess the burn depth. Although infrared imaging has been introduced as a noninvasive technique for assessing burn wounds, it has not been adapted clinically due to very low penetration capability, which means that infrared technology provides information about the surface area of the skin only.

Although the results obtained from terahertz imaging, optical coherence tomography, and infrared imaging technologies are promising, none of these technologies successfully assess the wound healing progress without the removal of dressing materials. The aim of this paper is to investigate the feasibility of using a passive millimeter-wave imaging system having a center frequency of 250 GHz to detect single and multiple burns under dressing materials without the necessity of dressings removal. This capability can be useful in the monitoring of the wound healing process under dressing materials.

The following sections in the paper are structured as follows: Section 2 describes the experimental methodology for scanning images and describes the porcine skin samples used in this research; Section 3 presents the experimental images obtained from the porcine skin samples before and after the application of localized heat treatment and with and without the presence of dressing materials; Section 4 discusses the images; and Section 5 draws the overall conclusions of the paper and highlights motivations for future directions.

## 2. Materials and Methods

### 2.1. Porcine Skin Samples

Seven fresh porcine skin samples were used in this research for the purpose of scanning images before and after the application of localized heat treatments and with and without the presence of dressing materials. The skin samples were taken directly after the animal was slaughtered for the purpose of commercial food and before the skin was washed. The measurements were conducted on the skin samples for a time of up to no longer than three hours after the slaughter. The samples were taken from pigs having ages ranging from five to nine months and average weights from 52 kg to 65 kg. The samples were taken from the back region of the animals. In general, the samples have a rectangular shape and average dimensions (length = 90 mm, width = 75 mm, and thickness = 4.0–11.0 mm).

### 2.2. Experimental Setup and Description

**Figure 2:**
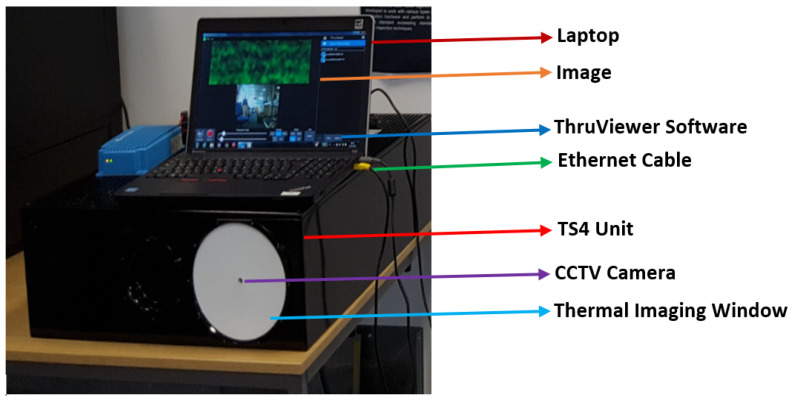
A ThruVision passive imager having a center frequency of 250 GHz comprising a TS4 unit, a CCTV camera, a thermal imaging window, and a laptop with a ThruViewer software. The distance between the imager and the target (i.e., porcine skin sample) is 800 mm. This distance was chosen since it is within the depth of field and the range of the imager (range = 1000 mm).

A ThruVision passive imager having a center frequency of 250 GHz and a bandwidth of 36 GHz (type: TS4, manufacturer: Digital Barriers, Abingdon, UK) [49] was used to scan images from the porcine skin samples. The imager is sensitive over the band 232–268 GHz and has a primary lens diameter of 175 mm and a measured depth of field of 200 mm (the span of range over which an object remains in focus). The imager has an operating wavelength of 1.2 mm and a spatial resolution of 5.5 mm, as summarized in Table 1. The imager consists of a TS4 unit and a laptop, as illustrated in Figure 2. The imager requires around 10 min of warm-up time. The TS4 unit is connected through an Ethernet cable to a laptop and the data are transferred from the TS4 unit to the laptop and vice versa. The images were displayed on the screen of the laptop using ThruViewer software version 7.2, and they were saved in screenshot format to be processed later using the Matlab program.

The measurements were conducted indoors in an anechoic environment (where there are no radiometric emissions from people or lower emissions from outdoors) at a room temperature of ~22 °C. The system output was assumed to be linear, as the lower color in the temperature scale represents the lower temperature (blue) and the higher color in the temperature scale (red) represents the higher temperature.

**Figure 3:**
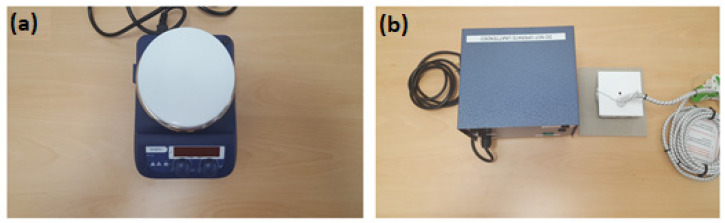
A digital hotplate used for heating the porcine skin samples (**a**) and a heat control device with metal plate used for performing burns on the porcine skin samples (**b**).

A digital hotplate illustrated in Figure 3a (type: LED digital hotplate magnetic stirrer, manufacturer: SciQuip Ltd., Wem, UK) with a temperature range of 280 °C was used to heat the porcine skin samples and to stabilize the skin surface temperature to ~35 °C. This temperature was chosen since it is similar to the in vivo surface temperature of the porcine skin ~35 °C as reported in [50,51]. The heat control metal plate (model number: SP2230-280H, manufacturer: SciQuip Ltd.) that is shown in Figure 3b consists of a temperature controller, thermocouple, and a square metal plate (50 mm × 50 mm). During the experimental work, the device was used to apply a contact burn after the plate was heated in the range of 100 °C to 140 °C and placed on the skin surface with constant pressure for a period of time of 60 s. Dressing materials (type: gauze burn dressing and light support bandage) were placed over the skin sample when required. All dressing materials were purchased from the pharmacy, and they were dry and removed from protective packaging prior to the measurements.

An infrared thermometer (type: N85FR, manufacturer: Maplin, Manchester, UK) with a temperature range of –50 °C to +550 °C and resolution of 0.1 °C was used to measure the skin surface temperature.

### 2.3. Methodology of Conducting the Experimental Work

This section discusses different methodologies used for scanning passive images of porcine skin samples. Images for porcine skin samples were scanned for the skin before and after the application of localized heat treatments with and without the presence of dressing materials.

#### 2.3.1. Methodology 1: Porcine Skin without Burns

The porcine skin sample was located over a digital hotplate and left to be heated and stabilized to 35 °C. Then, the sample was placed on a white polystyrene flat plate (length = 400 mm and width = 300 mm). The polystyrene plate combined with the sample was located vertically over a distance of 800 mm from the passive imager. Images of the skin were obtained using either screenshots or video recording options from the ThruViewer software version 7.2. The skin surface temperature of the sample was measured using an infrared thermometer.

**Figure 4:**
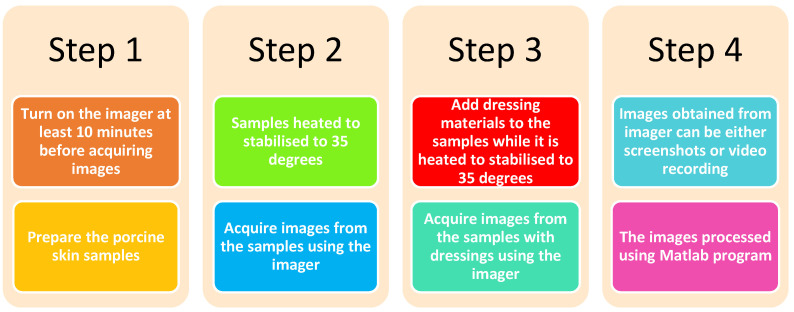
Methodology applied on porcine skin samples to acquire images for the skin with and without the presence of dressing materials.

In the case of skin with dressing materials, a similar methodology was applied to the sample. However, the dressing materials were located over the sample when the sample was located over the digital hotplate. This means that the temperature of the sample was stabilized to 35 °C with the presence of different types of dressing materials. This minimized the variation in the temperature of the skin as a result of adding extra dressing material layers. Figure 4 summarizes the methodology applied on porcine skin samples to obtain images for the skin with and without the presence of dressing materials.

#### 2.3.2. Methodology 2: Porcine Skin with Burns

The sample was located over a digital hotplate and left to be heated and stabilized to 35 °C. Then, contact burns were applied using a heat control metal plate. The plate was heated to 100 °C and placed directly on the skin surface for a period of 15 s with constant pressure. In this experimental work, single and multiple burns were applied on the sample. Then, the sample was placed on a white polystyrene flat plate. The plate combined with the sample was located vertically over a distance of 800 mm from the passive imager. Images of the skin were obtained using either screenshots or video recording options via the ThruViewer software version 7.2. The skin surface temperature of the sample was measured using an infrared thermometer.

**Figure 5:**
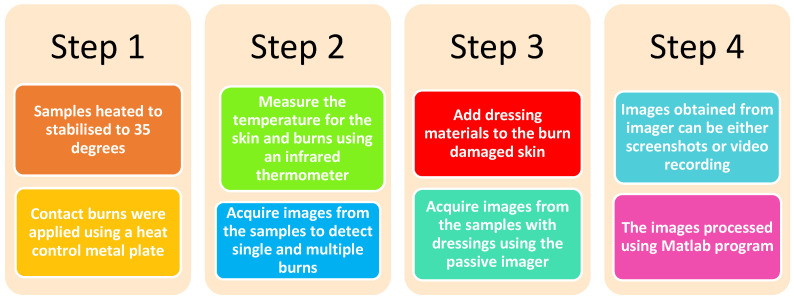
Methodology applied on burned skin samples to acquire images for the burned skin with and without the presence of dressing materials.

Then dressing materials were placed on the burn-damaged skin and images were obtained using the methodology described in Figure 5.

### 2.4. Methodology of Processing Images

Experimental images obtained via the ThruViewer software [49] were saved as screenshots in portable network graphic format (.png). This format was chosen as it allows the user to access the images without the presence of the ThruViewer software. The images were read using (imread) function implemented in the Matlab program. Then, color bars were inserted for assigning the temperature of different parts of the images (i.e., skin, burn, and background). The temperature measurements were made during the experimental work using an infrared thermometer. This type of thermometer was chosen as it can provide noncontact and precise measurements of the surface temperature of the skin.

## 3. Results

Experimental images for porcine skin samples in this section were divided into three parts: (1) images for unburned skin with and without the presence of dressing materials, (2) images for the burn-damaged skin without dressing materials, and (3) images for the burn-damaged skin with the presence of dressing materials.

### 3.1. Initial Measurements

**Figure 6:**
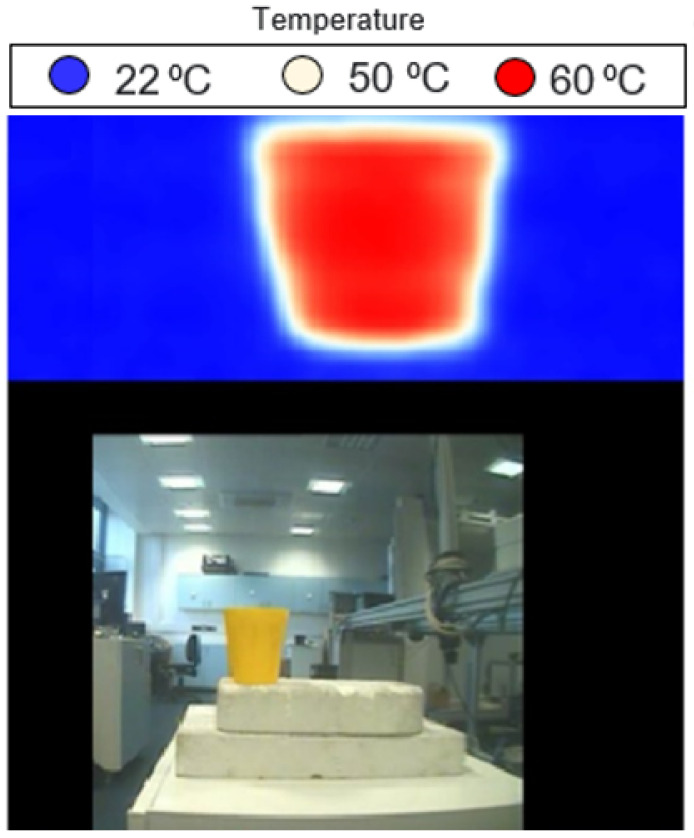
MMW image for a plastic cup having hot water inside.

The measurements presented in this section aim to identify the right match between the temperature measurements and the colors in the image. Therefore, a cup of hot water having a temperature of ~70 °C was located over a distance of 800 mm from the passive imager at an ambient temperature of ~22 °C as illustrated in Figure 6. The obtained image in Figure 6 shows the water (hot object) in red color and the background (ambient) in blue color and the plastic cup (which represents the point of contact to the environment) in light yellow color. Therefore, it is reasonable to assume that the lower color in the temperature bar represents the lower temperature (blue) and the higher color in the temperature bar (red) represents the higher temperature.

### 3.2. Unburned Skin

**Figure 7:**
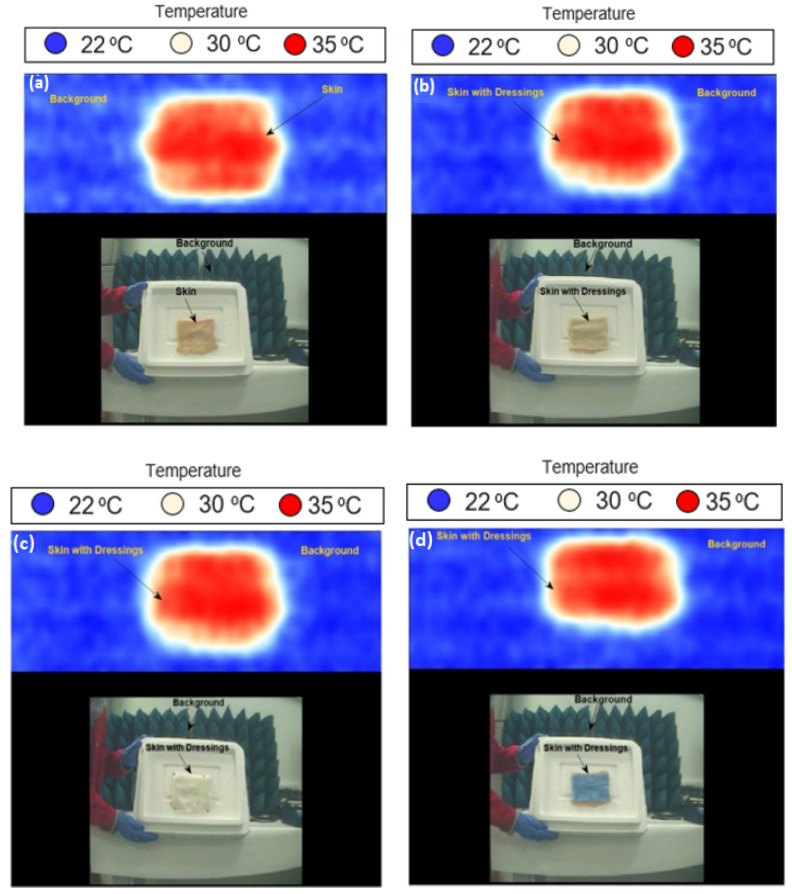
MMW images for porcine skin sample at 250 GHz; (**a**) represents the skin without dressings, (**b**) represents the skin with one-layer light support bandage, (**c**) represents the skin with 10-layer white gauze burn bandage, and (**d**) represents the skin with 4-layer blue gauze burn bandage. Each image has 229 (w) × 277 (h) pixels, and it takes 5 s to take a screenshot of an image.

Experimental images for porcine skin sample (length = 140 mm and width = 110 mm) without burns were performed using methodology 1 described in Section 2.3.1. The measurements were obtained at a room temperature of ~22 °C whilst the skin surface temperature was 35 °C. Different types of dressing materials were applied to the skin, and images were obtained and processed using the Matlab program, as illustrated in Figure 7.

Experimental images in Figure 7 indicate that the skin of the porcine sample is seen through different types of dressing materials having different thicknesses (thin dressings, such as a single-layer light support bandage in the Figure 7b, and thick dressings, such as a 10-layer gauze burn bandage in Figure 7c).

### 3.3. Skin with Single and Multiple Burns

**Figure 8:**
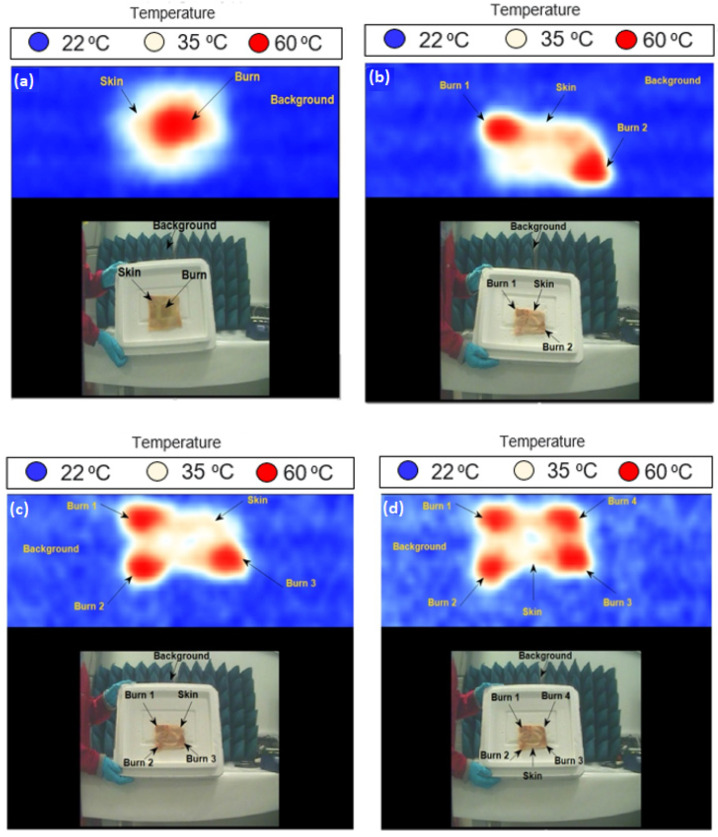
MMW images for porcine skin samples at 250 GHz; (**a**) represents the skin with one burn, (**b**) represents the skin with two burns, (**c**) represents the skin with three burns, and (**d**) represents the skin with four burns. Each image has 229 (w) × 277 (h) pixels, and it takes 5 s to take a screenshot of an image.

Images for porcine skin samples with single and multiple burns were performed using methodology 2 described in Section 2.3.2. The measurements were applied on two samples; sample 1 (length = 160 mm and width = 110 mm) and sample 2 (length = 110 mm and width = 100 mm). Images were obtained at a room temperature of ~22 °C and they were processed using the Matlab program, as illustrated in Figure 8.

Images in Figure 8 indicate that the passive MMW imaging system is capable of detecting burns and identifying the location of the burns (middle or edge), as illustrated in Figure 8a–d. The images also indicate that the passive imager is capable of distinguishing between the skin with burns (red color) and the skin without burns (white or yellow color). The radiation temperature of the skin ($T_{R}$) is directly proportional to the emissivity of the skin (ε) and the temperature of the skin (T), as $T_{R}=\varepsilon T$. Therefore, it is reasonable to assume that the radiation temperature of the skin with burns is equal to the thermodynamics temperature ~60 °C (or 333 K) as the emissivity of the porcine skin with burns is estimated to be ~1.0 at 250 GHz using the half-space model [12].

### 3.4. Skin with Burns and Dressing Materials

**Figure 9:**
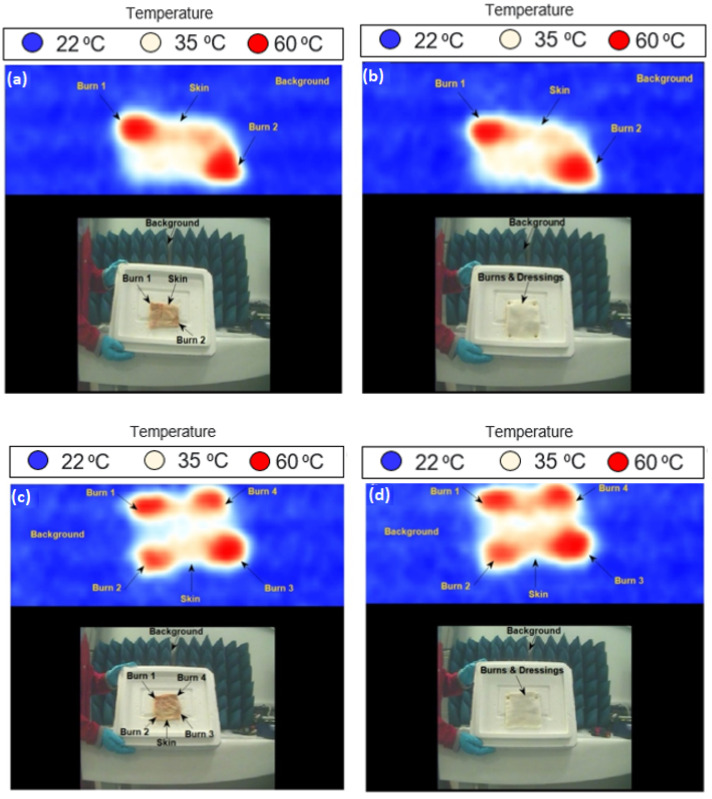
MMW images for porcine skin samples at 250 GHz; images in (**a**,**c**) represent the skin with burns and without dressing materials, whereas images in (**b**,**d**) represent the skin with burns and 10-layer gauze burn dressing materials. Each image has 229 (w) × 277 (h) pixels, and it takes 5 s to take a screenshot of an image.

Images for porcine skin samples with burns and dressing materials were performed using methodology 2 described in Section 2.3.2. The measurements were applied on two samples having multiple burns. Images were obtained at a room temperature of ~22 °C before and after the dressing materials were placed on the sample, as shown in Figure 9.

Images in Figure 9 indicate that thermal burns can be detected and identified under dressing materials without them being removed; similar images were obtained from the samples with dressings compared with those without dressings. The images also indicate that passive imaging systems can distinguish between the unburned skin (white or yellow color) and the burn-damaged skin (red color) with the presence of dressing materials, a capability that might be used for monitoring the burn wound healing progress.

## 4. Discussion

The measurements in this paper present images for the porcine skin samples taken from a passive imaging system having a center frequency of 250 GHz. The results obtained from these images indicate that it is feasible to detect features of the skin under dressing materials. These measurements suggest that a passive MMW imaging system might be an efficient tool for monitoring the wound healing progress under dressing materials without the necessity of dressings removal.

Experimental images obtained from porcine skin samples using the ThruVision passive imager before and after the application of localized heat treatments with and without the presence of dressing materials indicate that the PMMWI system can detect single and multiple burns under dressing materials. The measurements also indicate that the system can distinguish between the unburned skin (white, lower emissivity) and the burn-damaged skin (red, higher emissivity).

For in vivo scenarios, it is expected that similar images might be obtained, as there are differences in emissivity values between normal skin and burn-damaged skin [12]. These differences exist in all types of burns, and it is expected that these differences are becoming significant in serious injuries and burns situations where the removal of dressing materials might be uncomfortable and painful to the patients. These significant differences increase the chance of obtaining good images, as the images clearly distinguish between the burn and the unburned skin. It is also expected that the images obtained from a living organism might have different representations based on the degree of the burns and the presence of exudates and infections. An example of this is the second-degree burn with exudates that are expected to have a lower emissivity value compared with unburned skin. In this case, the image will show the normal skin in red color (higher emissivity) and the burn-damaged skin in white or yellow color (lower emissivity). However, for the third-degree burn injury, it is expected to see the normal skin in white or yellow color (lower emissivity) and the burn-damaged skin in red color (higher emissivity).

The measurements in this paper indicate that the use of passive imaging systems is feasible for detecting features of the skin under dressing materials. This capability might allow the system to detect changes on the skin surfaces during the wound healing process. The next step of progress in this area of research needs expansion of experimental investigations on patients; this manuscript and the results presented in it is aim to inform the medical community about the PMMWI technology in real medical practice.

## 5. Conclusions

Experimental images obtained from porcine skin samples over the band 232 GHz to 268 GHz indicate that single and multiple burns are observed under dressing materials. These results indicate that the PMMWI system can be used as a noncontact diagnostic technique for assessing dressed burn wounds as it can detect burns and the location of the burn under dressing materials. Images obtained from the ThruVision passive imager demonstrate the potential of the MMW technology for medical applications. The major outcome of this research is that it is possible to discriminate a burn wound from unburned skin under dressing materials using PMMWI. This highlights the opportunity that the healing of burn wounds may be assessed and monitored without the removal of dressing materials and, more importantly, in noncontact with the human body. As a plan for future work, it is recommended that passive images are acquired from patients having different degrees of burn injury. These would be analyzed to obtain a deeper understanding of the emissivity and the reflectivity of the skin and how these might change between patients and with the severity of the burn injuries. Furthermore, this will allow a comparison to be made between the human skin images and the porcine skin images conducted in this research, and this might help in identifying similarities and differences between the human skin and the porcine skin. The experimental setup presented in this research represents a proof-of-concept for initial capability demonstrations of detecting burns under dressings materials using PMMWI technology. To progress further we would require some developments to the experimental setup, such as adding an extra lens between the imager and the target object. The images with these follow-on developments are expected to have greater precision and convenience, offering a rapid and noninvasive (noncontact) diagnostic technique in a critical burns situation without the necessity of dressings removal.

## Figures and Tables

**Table 1:** A ThruVision passive imager specifications and parameters [49].

Imager Specification	Value
Frequency band	232–268 GHz
Center frequency	250 GHz
Band width	36 GHz
Depth of field	200 mm
Special resolution	5.5 mm
Operating wave length	1.2 mm
Lens diameter	175 mm
Field of view	150 mm (w) × 300 mm (h)
Frame rate	4 Hz

## Data Availability

Not applicable.
